# Tuberculosis patients with special clinical conditions treated with contezolid: three case reports and a literature review

**DOI:** 10.3389/fmed.2023.1265923

**Published:** 2023-12-14

**Authors:** Jun Wang, Liping Ma

**Affiliations:** Department of Tuberculosis, Beijing Chest Hospital, Beijing Tuberculosis and Thoracic Tumor Research Institute, Capital Medical University, Beijing, China

**Keywords:** contezolid, tuberculosis, oxazolidinone, linezolid, adverse reaction

## Abstract

**Background:**

Contezolid is a novel oxazolidinone antibacterial agent, but there have been no reports of any pertinent clinical studies for the treatment of tuberculosis (TB). This was the first report of three TB patients who were successfully treated with contezolid.

**Case presentation:**

Case 1 was TB complicated by myelosuppression syndrome. Case 2 was drug-resistant TB complicated by cirrhosis and anemia. Case 3 was drug-resistant TB complicated by liver transplantation that developed severe anemia after linezolid treatment. Following contezolid therapy, the three patients’ symptoms improved significantly, and no adverse reactions were observed. The chest computed tomography (CT) examination also indicated that the therapeutic effect of this anti-TB regimen was as expected.

**Conclusion:**

Contezolid showed good efficacy and fewer side effects in the treatment of TB. It may be a promising TB treatment.

## 1 Introduction

Tuberculosis (TB) represents a major threat to public health worldwide, and it ranks as the second leading cause of death among deaths caused by infectious diseases (1). Linezolid has long been the first-choice oxazolidinone antibiotic against multidrug-resistant or extensively drug-resistant tuberculosis (MDR/XDR-TB). Nevertheless, patients who use linezolid for a long time have serious adverse effects, leading to early discontinuation or dose reduction, which can compromise the efficacy of the drug and delay treatment (2, 3).

Contezolid is a new generation of oxazolidinone antibacterial agent with independent property rights in China. In contezolid, the A and B rings have a non-coplanar structure, which enhances the bacterial target binding, thus reducing the risk of drug resistance and killing bacteria more efficiently (4). It reduces the penetration of somatic cells and mitochondria, thus preserving the normal function of mitochondria and effectively reducing the side effects of myelosuppression (5). At present, it has completed phase III clinical trials in China and has been approved for marketing in China in 2021 (6). *In vitro* studies showed that contezolid had strong antibacterial activity against different types and sources of mycobacteria, and the intracellular bactericidal activity of contezolid against *Mycobacterium tuberculosis* was better than that of linezolid (7). In addition, the antibacterial activity of contezolid was also similar to that of linezolid in mouse models of TB (8).

Contezolid is a novel oxazolidinone antibacterial agent, but no relevant clinical study has been reported for the treatment of TB. We first present three cases of patients with TB who were treated with contezolid. Case 1 was TB, complicated by myelosuppression syndrome. Case 2 was drug-resistant TB, complicated by cirrhosis and anemia. Case 3 was drug-resistant TB, complicated by liver transplantation.

## 2 Case presentation

### 2.1 Case 1

#### 2.1.1 Case details

A 65 years-old man with a 2 years history of myelodysplasia was treated with leucogen tablets and blood enrichment nutri-gel. The patient complained of intermittent coughing and white sputum a year ago and was diagnosed with TB in our hospital. The patient has no family or genetic history of chronic diseases, cancer, or psychiatric disorders. The Xpert^®^ MTB/RIF (Xpert) assay showed positive PCR for bacterial DNA and no rpoB gene mutation. Sputum culture results showed a positive *M. tuberculosis* complex. Drug-susceptibility testing in TB showed that the patient was sensitive to isoniazid, levofloxacin, moxifloxacin, and rifampicin. A lung CT showed infectious lesions in both lungs, and the lumen of the bronchus dorsalis inferior lobe of the left lung is narrow. He was treated with isoniazid (0.3 g/qd), rifapentine (0.6 g/biw), pyrazinamide (1.5 g/qd), and ethambutol (1 g/qd) for 3 months but discontinued treatment due to leukocyte decline. He experienced coughing, aggravated sputum, a fever with a peak temperature of 38.5°C, and shortness of breath a week before admission. The chest CT was completed in other hospitals and showed multiple patchy shadows and cavitation in the left lung, as well as a pleural effusion on the left side. He was then transferred to our hospital for additional care.

#### 2.1.2 Admission diagnosis

**Figure 1:**
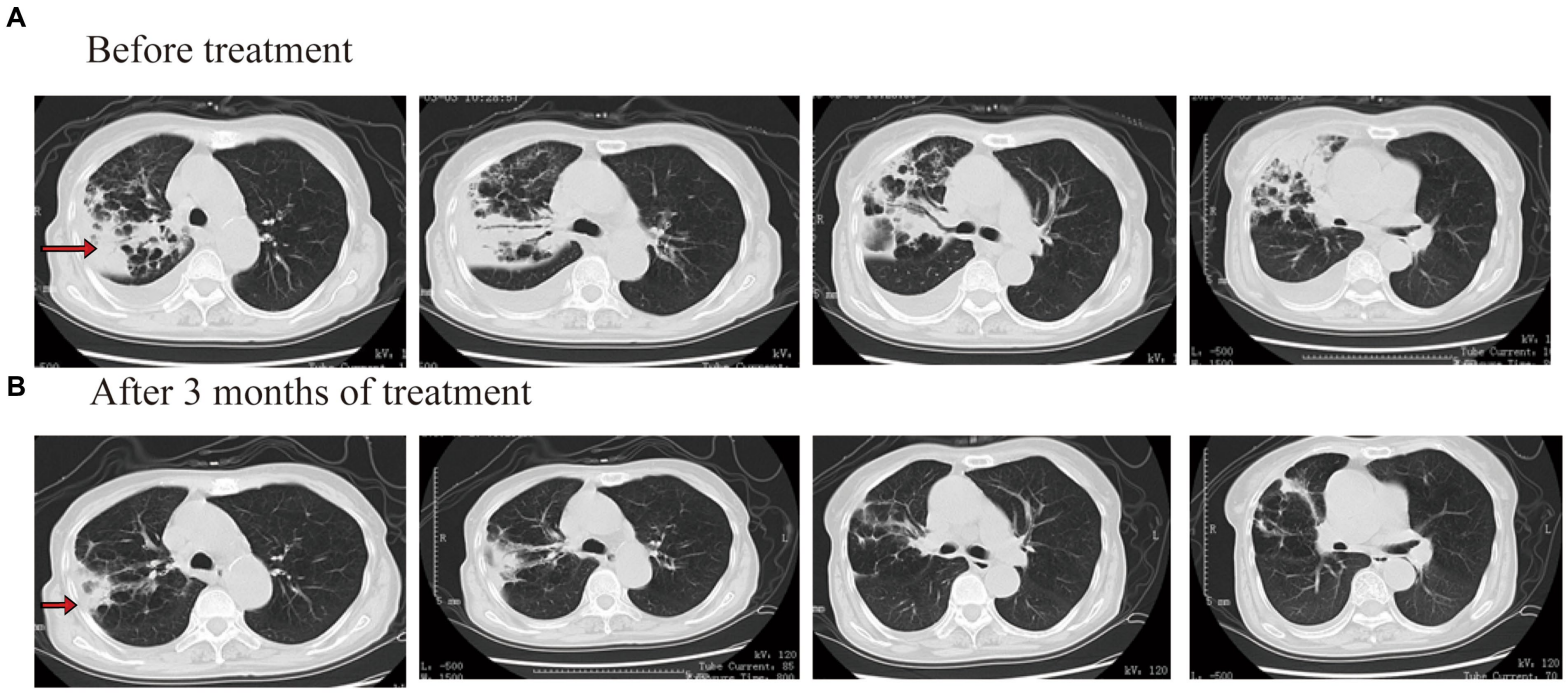
Chest CT scan images before and after treatment for case 1.

After admission to the hospital, laboratory tests showed that white blood count (WBC) was 1.51 × 10^9^/L, hemoglobin (HGB) was 81 g/L, platelets (PLT) was 47 × 10^9^/L, C-reactive protein (CRP) was 147.63 mg/L, procalcitonin (PCT) was 0.24 μg/L, and HBAC was 8.5%. No obvious abnormality was found in the blood biochemistry, galactomannan test (GM test), 1,3-β-D glucan test (G test), or blood autoimmune disease examination. *Mycobacterium* culture of sputum showed positive for acid-fast bacilli and positive for *M. tuberculosis* complex. The Xpert assay showed that the DNA-positive content of *M. tuberculosis* was low and there was no rpoB gene mutation. The results of *M. tuberculosis* resistance gene detection in sputum showed that the isoniazid resistance gene, the ethambutol resistance gene, and the quinolone resistance gene were negative. An enhanced CT examination of the chest revealed a caseous lesion in the upper right lung with cavities and a right pleural effusion (Figure 1A).

#### 2.1.3 Clinical course

**Figure 2:**
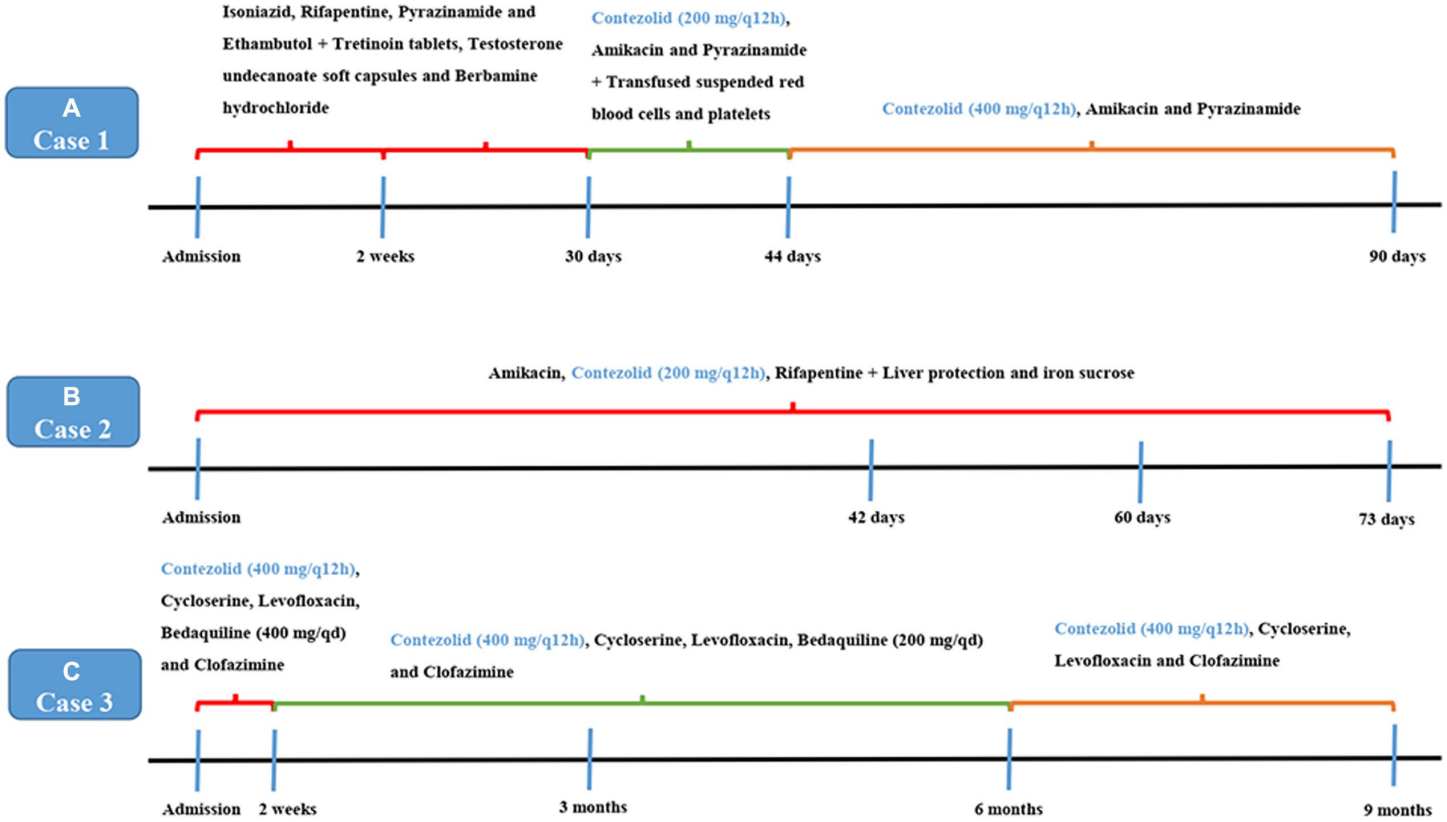
Administration process in cases 1–3.

After admission, isoniazid, rifapentine, pyrazinamide, and ethambutol were given anti-TB treatment again. Tretinoin tablets, testosterone undecanoate soft capsules, and berbamine hydrochloride were given treatment after a hematology consultation. After 2 weeks of treatment, the patient’s body temperature was lower than before, and his cough and sputum were better than before. The blood routine examination showed that WBC was 1.50 × 10^9^/L, HGB was 76 g/L, and PLT was 50 × 10^9^/L. After continuing the above treatment for 30 days, a reexamination of the blood routine showed that the WBC was 1.10 × 10^9^/L, HGB was 57 g/L, and PLT was 37 × 10^9^/L. Considering that the reduction of patients’ three cell lines was related to rifapentine and isoniazid, the use of anti-TB drugs was discontinued. Then, he was given contezolid (200 mg/q12h, orally), amikacin (0.4 g/qd, intravenously), and pyrazinamide (0.5 g/q8h, orally) treatment, while also being given transfusing suspended red blood cells and platelets. After 2 weeks of treatment (day 44), the patient’s body temperature returned to normal, and a reexamination blood routine showed that the WBC was 2.10 × 10^9^/L, HGB was 67 g/L, and PLT was 77 × 10^9^/L. Other therapies were not changed, and the dosage of contezolid was increased to 400 mg/q12h. There were also no more platelets or blood transfusions. On the 90th day of treatment, the patient’s temperature was normal, and he had no notable cough or sputum production. The blood routine examination showed that the WBC was 2.50 × 10^9^/L, HGB was 77 g/L, and PLT was 67 × 10^9^/L. The result of the Mycobacterium sputum culture was negative. The chest CT showed that the right lung consolidation shadow was significantly improved and the right pleural effusion was basically absorbed (Figure 1B). The administration process is shown in Figure 2A.

### 2.2 Case 2

#### 2.2.1 Case details

A 49 years-old woman was diagnosed with alcoholic cirrhosis 3 years ago and denied family or genetic history of chronic diseases, cancer, or psychiatric disorders. One year ago, the patient presented with a cough, white phlegm, night sweats, fatigue, and an occasional afternoon fever. She was admitted to a local hospital, and sputum smear results showed positive for acid-fast bacilli (2+). Due to the patient’s alcoholic cirrhosis, she was given isoniazid, ethambutol, rifapentine, and levofloxacin for anti-TB treatment for half a year. The patient felt that her health had improved and stopped taking the drug on her own. In December 2021, due to the recurrence of cough and sputum, she went to the local hospital. After 1 month of oral anti-TB drugs, she stopped taking anti-TB drugs again due to nausea and poor appetite. One week before admission, a cough and sputum appeared, with blood in the sputum, and the results of the sputum examination in the outpatient department of our hospital were positive for acid-fast bacilli (3+). Symptomatic hemostasis treatment was given, and hemoptysis was stopped. She was admitted to the hospital on 14 September 2022.

#### 2.2.2 Admission diagnosis

**Figure 3:**
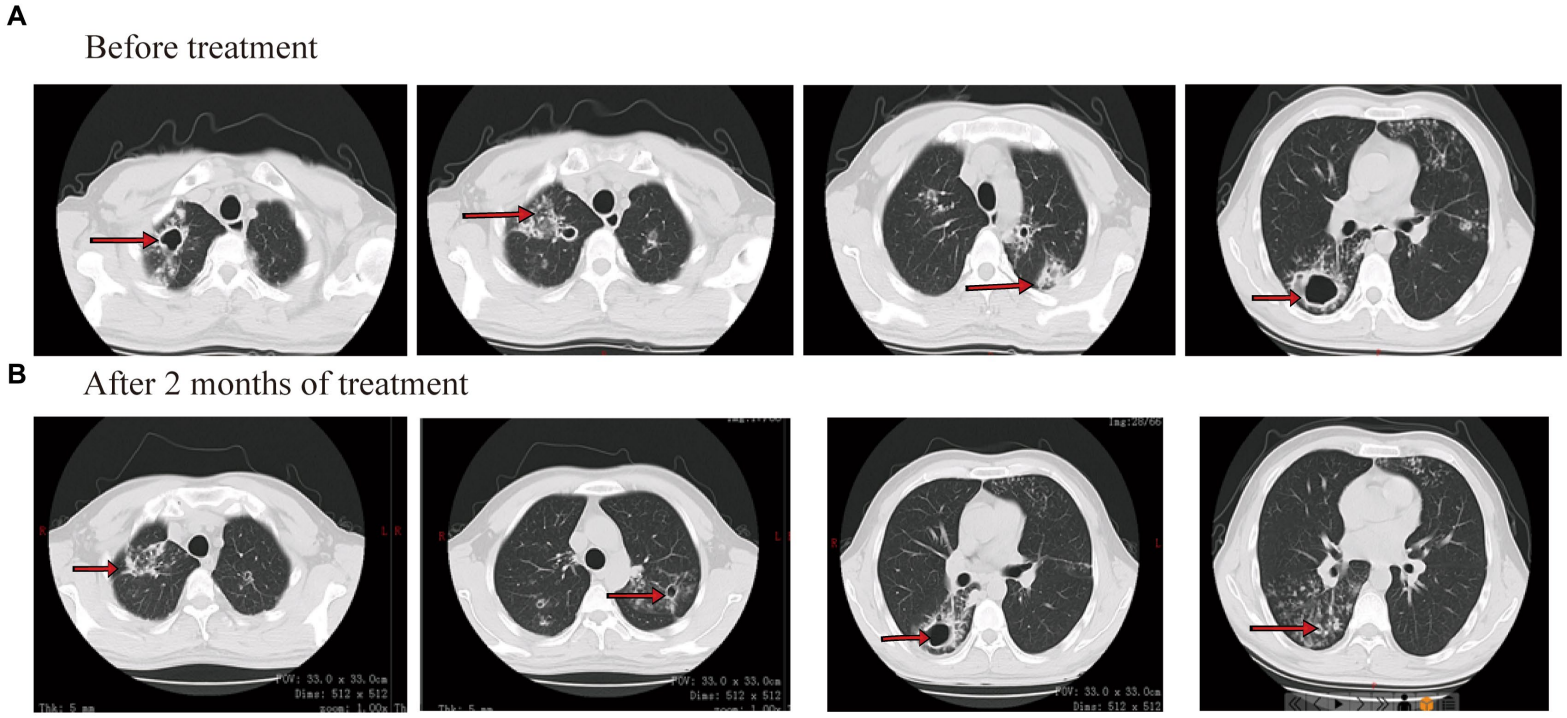
Chest CT scan images before and after treatment for case 2.

On laboratory findings, her WBC was 5.05 × 10^9^/L with a neutrophil ratio of 70.5%, HGB was 70 g/L, and PLT was 87 × 10^9^/L. Alanine aminotransferase (ALT) was 10 U/L, aspartate aminotransferase (AST) was 17 U/L, total bilirubin (TB) was 21.8 μmol/L, direct bilirubin (DB) was 7.6 μmol/L, and albumin (ALB) was 29 g/L. Smear results of sputum showed positive for acid-fast bacilli (4+). The Xpert results showed that the positive DNA content of TB bacteria was medium and there was no mutational resistance. Culture results of sputum showed *M. tuberculosis* complex positive. The drug sensitivity test for sputum showed that the patient was resistant to isoniazid, levofloxacin, and moxifloxacin and sensitive to rifampin. The results of drug resistance gene detection in *M. tuberculosis* showed mutations in the isoniazid resistance gene, ethambutol resistance gene, and quinolone resistance gene. CT showed that both lungs have multiple patches and cavities (Figure 3A).

#### 2.2.3 Clinical course

Considering the patient had cirrhosis and secondary anemia, pyrazinamide and linezolid were not used. She was given amikacin (0.4 g/qd, intravenously), contezolid (200 mg/q12h, orally), and rifapentine (0.45 g/biw, orally). Meanwhile, liver protection and iron sucrose were given to treat anemia. On 26 October 2022 (day 42 after treatment), sputum smear results showed negative for acid-fast bacilli. After 60 days of treatment, the blood routine showed WBC was 3.19 × 10^9^/L, HGB was 110 g/L, and PLT was 83 × 10^9^/L. The patient felt that her cough and sputum symptoms had significantly improved, that her body weight increased by 4 kg, and that she had no limb numbness. CT showed that both lung consolidation shadows were absorbed, and both pulmonary cavities were smaller than before (Figure 3B). On 26 November 2022 (day 73 after treatment), sputum smear results showed negative for acid-fast bacilli. The administration process is shown in Figure 2B.

### 2.3 Case 3

#### 2.3.1 Case details

A 59 years-old man underwent liver transplantation in 2012 due to hepatitis C and alcoholic liver disease. The patient developed a cough, yellow sputum, night sweats, fatigue, and fever one and a half years ago and denied a family or genetic history of chronic diseases, cancer, or psychiatric disorders. The chest CT showed multiple patchy shadows with cavities in both lungs. Sputum smear results showed positive for acid-fast bacilli (2+), diagnosed as TB. Anti-TB therapy with isoniazid, rifampicin, ethambutol, and pyrazinamide (HREZ) was given, and sputum acid-fast bacilli turned negative after 2 months, and anti-TB drugs were stopped due to rash after 5 months of treatment.

One year ago, he had a worse cough and sputum, accompanied by a fever. The Xpert results showed positive DNA of TB bacteria and mutations in the rpoB gene. The drug sensitivity test of the Lowenstein–Jensen medium culture in sputum showed resistance to isoniazid, rifampicin, and amikacin. He was admitted to our hospital for the first time in September 2020. Considering the patient had a history of cirrhosis and liver transplantation, he refused to use bedaquiline due to the failure of the cycloserine psychological assessment. He was treated with linezolid, moxifloxacin, and capreomycin for 14 days and stopped taking the drug by himself after discharge. In November 2020, he was orally given linezolid and levofloxacin. After 2 months of treatment, the patient visited the doctor due to fatigue. The HGB was 44 g/L, and anti-TB therapy was discontinued. In May 2021, he was given linezolid, levofloxacin, and faropenem for treatment. After 3 months of treatment, anemia and numbness of bilateral lower limbs occurred, which were considered to be related to linezolid, and anti-TB drugs were discontinued. Coughing and phlegm appeared again. Chest CT showed more lung lesions than before, and he was admitted to our hospital on 18 September 2021.

#### 2.3.2 Admission diagnosis

**Figure 4:**
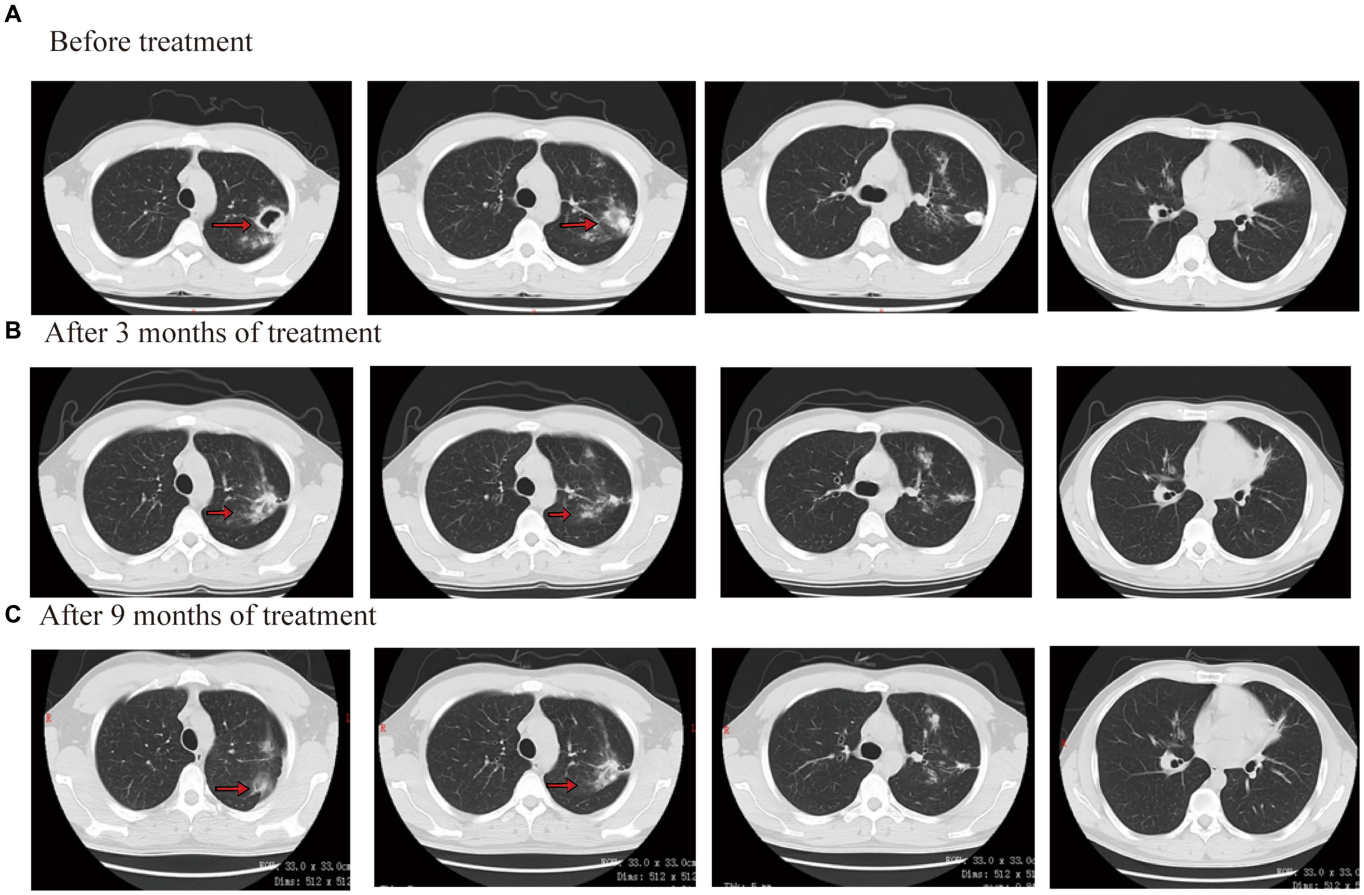
Chest CT scan images before and after treatment for case 3.

On laboratory findings, WBC was 5.05 × 10^9^/L with a neutrophil ratio of 70.5%, HGB was 86 g/L, and PLT was 226 × 10^9^/L. No obvious abnormality was found in the blood biochemistry, GM test, G test, or blood autoimmune disease examination. Sputum smear results showed positivity for acid-fast bacilli (2+). The Xpert results showed that the positive content of TB DNA was medium, and mutation resistance occurred. Sputum culture results showed *M. tuberculosis* complex positivity. The isoniazid resistance gene was positive, but the ethambutol and quinolone resistance genes were negative, according to the results of the drug resistance gene detection in *M. tuberculosis*. CT showed multiple patchy shadows in both lungs and a cavity in the left lung (Figure 4A).

#### 2.3.3 Clinical course

Considering that the patient underwent liver transplantation, some drugs were avoided as much as possible. The patient completed a cycloserine psychological assessment. Contezolid (400 mg/q12h, orally), cycloserine (0.25 g/bid, orally), levofloxacin (0.6 g/qd, orally), bedaquiline (400 mg/qd, orally), and clofazimine (100 mg/qd, orally) were given. Bedaquiline was changed to 200 mg/tiw after 2 weeks. The patient’s body temperature returned to normal, symptoms of cough and sputum improved, and numbness and pain in both lower limbs were relieved by approximately 70%. After 3 months of treatment outside the hospital, the lesions were clearly absorbed and improved. The CT scan showed that both lung consolidation shadows had been absorbed and the left lung cavity had closed (Figure 4B). In November 2021, the result of the *Mycobacterium* sputum culture was negative. The WBC was 4.56 × 10^9^/L with a neutrophil ratio of 75.5%, HGB was 116 g/L, and PLT was 138 × 10^9^/L. Bedaquiline was discontinued after 6 months and continued to be treated with contezolid, cycloserine, levofloxacin, and clofazimine. After 3 months of treatment, the patient’s body temperature was normal, there was no obvious cough or sputum, and the numbness and pain in both lower limbs were relieved. In March 2022, the result of the *Mycobacterium* sputum culture was negative. In June 2022, CT showed that both lung diseases were smaller than before (Figure 4C). The administration process is shown in Figure 2C.

## 3 Discussion

TB is the leading cause of infectious mortality in the world. The World Health Organization Global Tuberculosis Report 2022 shows that the number of people newly diagnosed and reported with TB globally decreased by 18% in 2019–2020 after a large increase in 2017–2019. In 2021, the number of people with TB worldwide has increased to 6.4 million. A total of 1.6 million people died from TB in 2021, compared with 1.5 million in 2020 (9). Patients with MDR/XDR-TB require longer treatment times than those with drug-susceptible TB and have a low success rate (10). It remains a global impediment to the anti-TB strategy.

Linezolid has been used for the treatment of MDR/XDR-TB (11, 12). However, despite its excellent efficacy, serious adverse events have been observed, which include anemia, neutropenia, myelosuppression, thrombocytopenia, peripheral neuropathy, optic neuropathy, lactic acidosis, pancreatitis, and rhabdomyolysis (13–16). With the increase in dosage, more and more adverse events are being reported. According to the Expert Consensus on anti-TB Treatment of linezolid (17), 15.81% of patients stopped using linezolid due to serious adverse reactions, 32.93% of patients suffered from myelosuppression (anemia, leukopenia, and thrombocytopenia), and 29.92% of patients suffered from peripheral neuritis or optic nerve damage. A highly successful NIX-TB trial in South Africa revealed that after treatment with the bedaquiline-pretomanid-linezolid regimen, 81% of patients developed peripheral neuropathy, and 48% developed myelosuppression (18). In the follow-up ZeNix trial, reducing the daily dose of linezolid still caused peripheral neuropathy in 24% of patients (19). In case 3 presented here, linezolid was discontinued due to adverse drug reactions such as anemia. Therefore, we have carried out the exploration of new drugs and new regimens and selected contezolid with better safety as an alternative.

Contezolid is a novel oxazolidinone antibiotic that replaces the morpholine in linezolid with a piperidinone (20). Drug interactions must be taken into consideration when using many medications because critically ill infected individuals frequently have low organ function and rapid illness progression. Fortunately, contezolid does not interact with the majority of popular medications and is ideally suited to combination therapy because it is not processed by the CYP liver drug enzyme system but rather by the yellow monooxygenase pathway, which has less effect on the monoamine oxidase system (6). Contezolid is well suited for combination therapy due to its specific metabolic pathway. In phase III clinical trial, contezolid demonstrated a significantly lower incidence of leucopenia and thrombocytopenia than linezolid in adults with complicated skin and soft tissue infections (21). An early study reported a case of tuberculous pleurisy in an 87 years-old woman with thrombocytopenia caused by isoniazid and linezolid during treatment. After 4 weeks of treatment with contezolid plus cycloserine, the results were good (22). Only a few studies have been reported on the effect of contezolid on TB. One study evaluated the *in vitro* and *in vivo* activities of contezolid against TB. Its activity in a murine tuberculosis model was also similar to that of linezolid (8). However, no clinical studies on contezolid against TB have been reported.

The strength of this study was that it first introduced the efficacy and safety of contezolid to three special TB patients. The follow-up time for three patients was 2 to 9 months. During treatment with contezolid, hemoglobin and platelets did not decrease significantly, which improved the hematological toxicity caused by linezolid, especially the reduction of platelet levels. The chest CT examination also indicated that the therapeutic effect of this anti-TB regimen was in line with expectations. Throughout the course of treatment, the patient did not show any discomfort or dissatisfaction with the treatment with contezolid. They were able to be proactive and cooperate to complete the treatment, and they believed that contezolid could play an important role in the treatment of tuberculosis. Additionally, the manuscript has some restrictions. It is challenging to explain the therapeutic effect of a single antibiotic because the patient received contezolid and other antibiotics at the same time. Despite the fact that this trial included three patients, contezolid needs to be studied in a wider population.

## 4 Conclusion

Contezolid showed good efficacy and fewer side effects in the treatment of TB. The inclusion of contezolid may make TB treatment regimens more effective and less toxic than linezolid. This regimen is a promising treatment strategy for TB. However, large-scale randomized controlled trials still need to be conducted to determine the efficacy and safety of contezolid in treating TB.

## Data availability statement

The original contributions presented in the study are included in the article/supplementary material, further inquiries can be directed to the corresponding author.

## Ethics statement

Written informed consent was obtained from the individual(s) for the publication of any potentially identifiable images or data included in this article.

## Author contributions

JW: Conceptualization, Data curation, Formal analysis, Project administration, Resources, Writing – original draft, Writing – review & editing. LM: Conceptualization, Data curation, Formal analysis, Project administration, Resources, Writing – original draft, Writing – review & editing.
